# Stiffness on shear wave elastography as a potential microenvironment biomarker for predicting tumor recurrence in HBV-related hepatocellular carcinoma

**DOI:** 10.1186/s13244-023-01505-7

**Published:** 2023-09-12

**Authors:** Xian Zhong, Haiyi Long, Lili Chen, Yuhua Xie, Yifan Shi, Jianyun Peng, Ruiying Zheng, Liya Su, Yu Duan, Xiaoyan Xie, Manxia Lin

**Affiliations:** 1 Department of Ultrasound, The First Affiliated Hospital of Sun Yat-Sen University, 58 Zhongshan Second Road, Guangzhou, 510080 China; 2 Department of Pathology, The First Affiliated Hospital of Sun Yat-Sen University, 58 Zhongshan Second Road, Guangzhou, 510080 China

**Keywords:** Hepatocellular carcinoma, Two-dimensional shear wave elastography, Microenvironment, Tumor recurrence, Stiffness

## Abstract

**Background:**

To explore the pathologic basis and prognostic value of tumor and liver stiffness measured pre-operatively by two-dimensional shear wave elastography (2D-SWE) in hepatitis B virus (HBV)-related hepatocellular carcinoma (HCC) patients who undergo hepatic resection.

**Methods:**

A total of 191 HBV-infected patients with solitary resectable HCC were prospectively enrolled. The stiffness of intratumoral tissue, peritumoral tissue, adjacent liver tissue, and distant liver tissue was evaluated by 2D-SWE. The correlations between stiffness and pathological characteristics were analyzed in 114 patients. The predictive value of stiffness for recurrence-free survival (RFS) was evaluated, and Cutoff Finder was used for determining optimal cut-off stiffness values. Cox proportional hazards analysis was used to identify independent predictors of RFS.

**Results:**

Pathologically, intratumoral stiffness was associated with stroma proportion and microvascular invasion (MVI) while peritumoral stiffness was associated with tumor size, capsule, and MVI. Adjacent liver stiffness was correlated with capsule and liver fibrosis stage while distant liver stiffness was correlated with liver fibrosis stage. Peritumoral stiffness, adjacent liver stiffness, and distant liver stiffness were all correlated to RFS (all *p* < 0.05). Higher peritumoral stiffness (> 49.4 kPa) (HR = 1.822, *p* = 0.023) and higher adjacent liver stiffness (> 24.1 kPa) (HR = 1.792, *p* = 0.048) were significant independent predictors of worse RFS, along with tumor size and MVI. The nomogram based on these variables showed a C-index of 0.77 for RFS prediction.

**Conclusions:**

Stiffness measured by 2D-SWE could be a tumor microenvironment and tumor invasiveness biomarker. Peritumoral stiffness and adjacent liver stiffness showed important values in predicting tumor recurrence after curative resection in HBV-related HCC.

**Clinical relevance statement:**

Tumor and liver stiffness measured by two-dimensional shear wave elastography serve as imaging biomarkers for predicting hepatocellular carcinoma recurrence, reflecting biological behavior and tumor microenvironment.

**Key points:**

• Stiffness measured by two-dimensional shear wave elastography is a useful biomarker of tumor microenvironment and invasiveness.

• Higher stiffness indicated more aggressive behavior of hepatocellular carcinoma.

• The study showed the prognostic value of peritumoral stiffness and adjacent liver stiffness for recurrence-free survival.

• The nomogram integrating peritumoral stiffness, adjacent liver stiffness, tumor size, and microvascular invasion showed a C-index of 0.77.

**Graphical Abstract:**

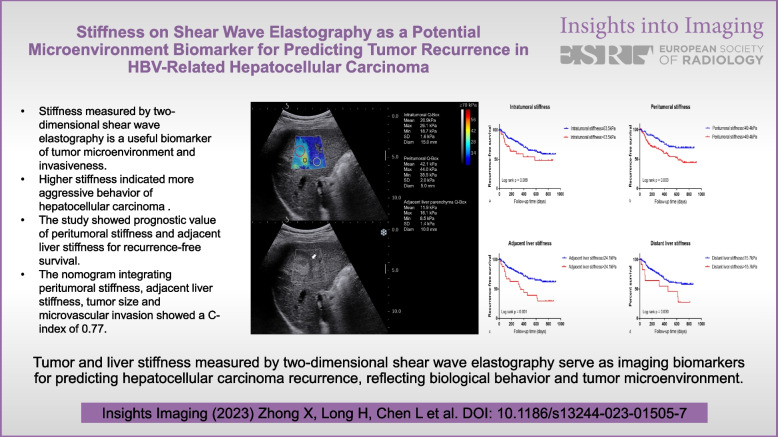

**Supplementary Information:**

The online version contains supplementary material available at 10.1186/s13244-023-01505-7.

## Background

Hepatocellular carcinoma (HCC) is the fifth most common malignancy and the third leading cause of cancer-related death worldwide [1]. Surgical resection is the first-line therapy in patients with solitary tumors and well-preserved liver function. However, recurrence after surgical resection is common, with 5-year recurrence rates reaching 70% [2]. In addition to tumor size, tumor number, and liver function, the microenvironment and biologic behavior of tumors are also considered as important prognostic factors [3, 4].

The tissue stiffness reflects the mechanical properties and characterizes the complex interactions between tumor cells and extracellular matrix (ECM). The findings from various studies indicate that higher matrix stiffness promotes proliferation and chemotherapeutic resistance [5], upregulates VEGF expression [6], and enhances stemness [7] in HCC, which suggests the value of tissue stiffness to function as an integrative biomarker for HCC aggressiveness and prognosis [8]. Studies have demonstrated the value of tumor stiffness for predicting tumor recurrence following hepatic resection [9–11], showing a positive correlation between higher tumor stiffness and increased recurrence rates. It was reported that higher matrix stiffness could trigger epithelial-mesenchymal transition in HCC and facilitate HCC invasion and metastasis, which suggested the role of tumor stiffness in characterizing tumor aggressiveness [5, 12]. Besides, liver stiffness was also reported to be an independent predictor of recurrence in HCC after surgical resection or radiofrequency ablation [13–17]. The liver stiffness, mainly related to the degree of liver fibrosis, has been proved to be involved in both carcinogenesis and progression of HCC [18–20]. Therefore, both tumor stiffness and liver stiffness could serve as comprehensive biomarkers for evaluating the tumor microenvironment, offering the possibility of being a prognostic marker for HCC recurrence.

The two-dimensional shear wave elastography (2D-SWE) technique is an ultrasound-based technique for real-time visualization of soft tissue’s viscoelastic properties by measuring the speed of shear waves generated using acoustic radiation force [21]. The performance for assessment of diffuse liver disease by 2D-SWE has been confirmed [21, 22].

Several studies have also demonstrated the utility of 2D-SWE for the evaluation of tissue stiffness and for diagnosis or prognosis of focal liver lesions (FLLs) [23–26], and for characterizing tumor microenvironment and revealing the behavior of tumor-stroma interactions in HCC [27]. It may provide important tumor biologic, pathological, and ultimately prognostic information for HCC patients. Previous studies have reported the prognostic value of stiffness measured by magnetic resonance elastography, transient elastography, or acoustic radiation force impulse elastography for HCC recurrence [9–11, 13–17]. However, it remains unclear whether tumor stiffness and liver stiffness measured by 2D-SWE can also be used as prognostic markers for HCC recurrence after curative treatment. Furthermore, the pathological basis of the stiffness in HCC is also unknown.

Therefore, the hypothesis of this study is that tumor stiffness and liver stiffness measured by 2D-SWE can reflect the biological behavior and tumor microenvironment, thus serving as an imaging biomarker for predicting the prognosis of HCC. This study aimed to assess the pathological basis of tumor and liver stiffness and to evaluate the potential utility of tissue stiffness measured by 2D-SWE for predicting the recurrence of hepatitis B virus (HBV)-related HCC patients after hepatic resection, with a focus on combining tumor stiffness and liver stiffness for prediction.

## Materials and methods

### Patients

**Fig. 1:**
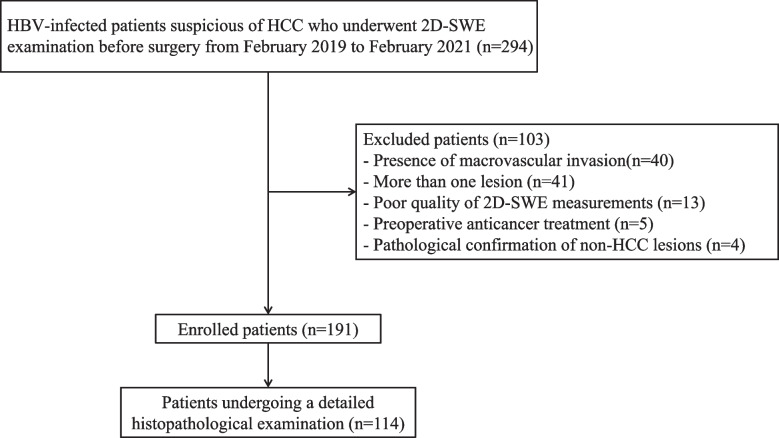
Flow chart of the enrolled patients in our study

This prospective study was approved by the ethics committee of the First Affiliated Hospital of Sun Yat-Sen University. Written informed consents were obtained from all patients before enrollment. HBV-infected patients with solitary HCC who underwent surgical resection from February 2019 to February 2021 were consecutively included in this study according to the inclusion and exclusion criteria. The diagnosis of HCC before surgery was based on the American Association for the Study of Liver Diseases (AASLD) guideline [2]. Inclusion criteria included the following: (1) patients aged 18–80 years; (2) patients with solitary resectable HCC; (3) performance status Eastern Cooperative Oncology Group score 0–1. Exclusion criteria included the following: (1) poor quality of 2D-SWE image data (e.g., the elastography color map was less than 75% filled); (2) the lesions received local or systematic anti-tumor therapies before 2D-SWE examination or surgery; (3) Pathological confirmation of non-HCC lesions. Figure 1 shows the patient recruitment process.

### Ultrasound data acquisition

Both conventional ultrasound and 2D-SWE examinations were performed within one week before surgery. All examinations were performed by an Aixplorer Ultrasound system (SuperSonic Imagine, France) equipped with an SC6-1 convex probe by a radiologist with more than 10 years of ultrasound experience and more than 3 years of experience in liver 2D-SWE examination (M.X.L.).

After overnight fasting for at least 8 hours, the patient was placed in the supine position with the right arm in maximal abduction. 2D-SWE examination was performed for both tumor and liver according to the European Federation of Societies for Ultrasound in Medicine and Biology (EFSUMB) guideline [21]. For 2D-SWE examination of the tumor and adjacent liver, a B-mode ultrasound scan was first performed to determine the maximum cross-section of the tumor and adjacent liver parenchyma of at least 1cm. Then the ultrasound mode was switched to elasticity imaging mode. A 2D-SWE window of 4 cm × 3 cm was placed at a depth of 1–8 cm beneath the liver capsule and the scale was 70 kPa [28]. For tumors < 3 cm, the 2D-SWE window overlaid the tumor and adjacent liver parenchyma of at least 1.0 cm [29]. For tumors ≥ 3 cm, the 2D-SWE window overlaid a part of the tumor and adjacent liver parenchyma of at least 1.0 cm. The patients were asked to hold their breath for several seconds during quiet breathing and five sequential images were obtained when the color map filled more than 75% of the 2D-SWE window and the signal stabilized for a few seconds [30]. For 2D-SWE examination of distant liver tissue, a B-mode ultrasound scan was first performed to locate a well-visualized liver area free of large vessels (diameter > 3 mm) and at least 5 cm away from the lesion margin. Areas in the right anterior lobe of the liver were preferred if available. Then the ultrasound mode was switched to elasticity imaging mode with the scale of 40 kPa. A 2D-SWE window of 4 cm × 3 cm was placed at a depth of 1.5–2 cm beneath the liver capsule. Patients were asked to hold their breath for a few seconds to obtain five sequential images once the elastography signal became stable and the color filling in the sampling frame reached 75%. The median and interquartile range (IQR) of five sequential acquisitions (in kilopascals) was calculated. An IQR/median < 30% was considered successful.

### Image analysis

The B-mode ultrasound and 2D-SWE images were analyzed by one radiologist (X.Z.) with more than 5 years of experience in ultrasound, who was unaware of the pathological results. Evaluations of B-mode ultrasound images were performed from the PACS system. The 2D-SWE image analysis was performed on the machine using the built-in ROI (Q-box) whose size and position were adjustable.

**Fig. 2:**
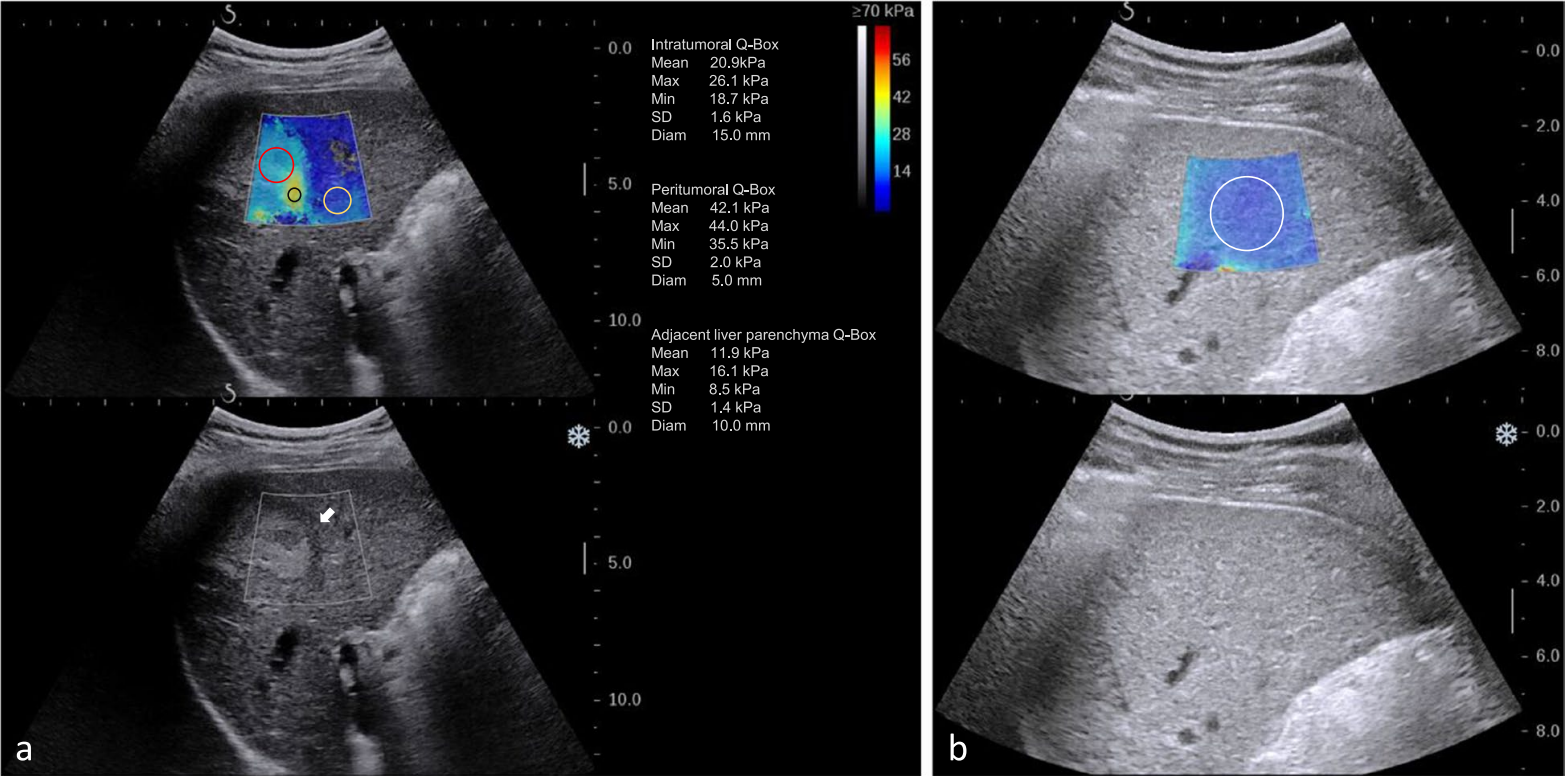
Elasticity measurement of 2D-SWE images. **a** Regions of interest (ROI) for measurement of the stiffness of intratumoral tissues (red ROI), peritumoral tissues (black ROI), and adjacent liver parenchyma (yellow ROI). **b** ROI for distant liver parenchyma (white ROI)

Evaluations of tumor B-mode ultrasound images included tumor size, shape, boundary, and presence of hypoechoic halo. Evaluations of 2D-SWE images included quantitative stiffness of both intratumoral and peritumoral tissue, as well as adjacent liver parenchyma and distant liver parenchyma. An intratumoral Q-box was placed inside the tumor to cover the tumor area as much as possible for intratumoral stiffness [26]. A peritumoral Q-box of about 5 mm was placed in the tumor border with the highest stiffness [31, 32]. An adjacent liver parenchyma Q-box of about 1cm was placed in adjacent liver parenchyma [26] (Fig. 2a). A distant liver parenchyma Q-box of about 2 cm was placed in distant liver parenchyma (Fig. 2b). The mean values (Emean) of Young’s modulus in these four areas were recorded. The median values of Emean in five sequential images were used for further analysis.

### Clinical data collection and pathological examination

Preoperative patient characteristics and laboratory data were collected within one week ahead of surgery. Due to the unavailability of some pathological specimens, the pathological specimens of 114 patients from February 2019 to January 2020 were reviewed by a pathologist (L.L.C.) with more than 10 years of experience in HCC pathology, without knowing the patient’s clinical data and ultrasound results. The specimens were sampled according to the 7-point baseline sampling protocol [33]. Information about Edmondson-Steiner grade, proportion of stroma, the presence and proportion of tumor necrosis, the presence of tumor capsule, degree of peritumoral lymphocytic reaction, microvascular invasion (MVI) of HCC, and grade of liver fibrosis were evaluated using a microscope on 4-μm paraffin-embedded histological sections with hematoxylin and eosin staining. The presence of cirrhosis was defined as S4 according to the Scheuer liver fibrosis staging system by pathological examination [34]. Definitions of the other pathological characteristics can be found in the Supplementary material.

### Follow-up

Patients were consistently followed up after liver resection at intervals of 3 to 6 months based on serum alpha-fetoprotein and imaging examination (contrast-enhanced computed tomography or contrast-enhanced magnetic resonance imaging). All patients were under anti-viral treatment after resection. All patients were followed up until September 2021. The mean period of follow-up was 14.2 ± 9.2 months (range 1.5–30.7 months). Recurrence-free survival (RFS) was calculated from the date of surgical resection to the tumor recurrence (local recurrence, new intrahepatic tumor, vascular invasion, or distant organ metastasis). RFS was censored at the date of death or of the last follow-up visit for recurrence-free patients.

### Statistical analysis

Statistical analyses were performed by using SPSS, version 20.0, and R4.1.2. The Student’s *t*-test or the Mann-Whitney test, as appropriate, was used to compare continuous variables in recurrence and non-recurrence groups. The *χ*^2^ test was used to compare categorical variables. The elasticity values of different pathological manifestations were compared by using the Mann-Whitney test. An online tool Cutoff Finder was used to determine the optimal cut-off elasticity values for predicting RFS [35]. The elasticity values were dichotomized based on the optimal cut-off values. Univariable and multivariable analyses were performed to determine the significant clinical, ultrasound, and pathological factors for RFS prediction. The survival curves were generated using the Kaplan–Meier method and the log-rank test was applied to compare the differences between groups. After the univariable Cox proportional hazards model was applied to each variable, the variables with a *p* value less than 0.05 entered multivariate analysis to identify independent predictors for RFS based on stepwise Cox proportional hazards regression. Schoenfeld residuals were used to check the proportional hazards assumption of the Cox proportional hazards model and a stepwise Cox regression was used to prevent multicollinearity. A nomogram was constructed based on independent predictors. The discrimination performance was quantified by concordance index (C-index) and area under the receiver operating characteristic curve (AUC) at 0.5 years, 1 year, and 2 years. The calibration performance was determined by calibration curve analysis. All statistical tests were two-tailed and a *p* value < 0.05 was considered a statistically significant difference.

## Results

### Baseline characteristics

A total of 191 HBV-infected patients with single HCC who underwent surgical resection were prospectively enrolled in this study, including 173 males and 18 females with a median age of 55.0 (47.0–64.0) years (Fig. 1).

During the follow-up, HCC recurrence was observed in 66 (34.6%) patients. The baseline characteristics of the recurrence group, non-recurrence group, and all patients were summarized in Table 1. There were significant differences in tumor size (*p* < 0.001), presence of MVI (*p* < 0.001), AFP level (*p* = 0.006), AST level (*p* = 0.005), GGT level (*p* = 0.007), and peritumoral stiffness (*p* = 0.020) between the recurrence group and non-recurrence group.

**Table 1:** Clinical and ultrasound characteristics of patients included in this study

Characteristic	All patients (*n* = 191)	Non-recurrence group (*n* = 125)	Recurrence group (*n* = 66)	*p* value
Age (year)^a^	55.0 (47.0–64.0)	55.0 (47.5–64.0)	55.0 (46.5–62.5)	0.346
Sex				0.354
Male	173 (90.6)	115 (92.0)	58 (87.9)	
Female	18 (9.4)	10 (8.0)	8 (12.1)	
Tumor size (cm)^a^	5.3 (3.6–7.8)	4.7 (3.3–6.5)	7.7 (5.0–10.5)	< 0.001
BCLC stage				0.205
Very early stage (0)	3 (1.6)	3 (2.4)	0 (0.0)	
Early stage (A)	188 (98.4)	122 (97.6)	66 (100.0)	
Child-Pugh class				0.949
A	185 (96.9)	121 (96.8)	64 (97.0)	
B	6 (3.1)	4 (3.2)	2 (3.0)	
Edmondson-Steiner grade				0.099
Grade I–II	88 (46.1)	63 (50.4)	25 (37.9)	
Grade III–IV	103 (53.9)	62 (49.6)	41 (62.1)	
Microvascular invasion				< 0.001
Absent	110 (57.6)	84 (67.2)	26 (39.4)	
Present	81 (42.4)	41 (32.8)	40 (60.6)	
Cirrhosis of background liver				0.310
Absent	139 (72.8)	88 (70.4)	51 (77.3)	
Present	52 (27.2)	79 (29.6)	15 (22.7)	
AFP				0.006
≤ 20 U/L	90 (47.1)	68 (54.4)	22 (33.3)	
> 20 U/L	101 (52.9)	57 (45.6)	44 (66.7)	
TBIL				0.816
≤ 17.1 µmol/L	138 (72.3)	91 (72.8)	47 (71.2)	
> 17.1 µmol/L	53 (27.7)	34 (27.2)	19 (28.8)	
ALB				0.244
< 35 g/L	164 (85.9)	110 (88.0)	54 (81.8)	
≥ 35 g/L	27 (14.1)	15 (12.0)	12 (18.2)	
ALT				0.751
≤ 40 U/L	133 (69.6)	88 (70.4)	45 (68.2)	
> 40 U/L	58 (30.4)	37 (29.6)	21 (31.8)	
AST				0.005
≤ 40 U/L	116 (60.7)	85 (68.0)	31 (47.0)	
> 40 U/L	75 (39.3)	40 (32.0)	35 (53.0)	
GGT				0.007
≤ 50 U/L	92 (48.2)	69 (55.2)	23 (34.8)	
> 50 U/L	99 (51.8)	56 (44.8)	43 (65.2)	
HBV-DNA				0.178
≤ 100 IU/mL	88 (46.1)	62 (49.6)	26 (39.4)	
> 100 IU/mL	103 (53.9)	63 (50.4)	40 (60.6)	
Intratumoral stiffness (kPa) ^a^	32.1 (20.6–49.8)	31.5 (10.5–48.1)	35.7 (23.1–53.1)	0.179
Peritumoral stiffness (kPa) ^a^	48.3 (30.4–62.7)	43.8 (28.2–60.5)	51.9 (39.4–66.3)	0.020
Adjacent liver stiffness (kPa) ^a^	12.5 (9.5–18.7)	12.7 (9.5–18.3)	12.2 (9.7–21.4)	0.600
Distant liver stiffness (kPa) ^a^	8.8 (7.0–11.4)	8.5 (6.8–10.6)	9.2 (7.3–12.0)	0.123

*AFP*, alpha-fetoprotein; *TBIL*, total bilirubin; *ALB*, albumin; *ALT*, alanine aminotransferase; *AST*, aspartate transaminase; *GGT*, gamma-glutamyl transferase; *HBV*, hepatitis B virus

Unless otherwise indicated, data are shown as number of patients, with the percentage in parentheses

^a^Data are shown as median (interquartile range)

### Pathological basis of intratumoral, peritumoral, adjacent liver and distant liver elasticity

**Fig. 3:**
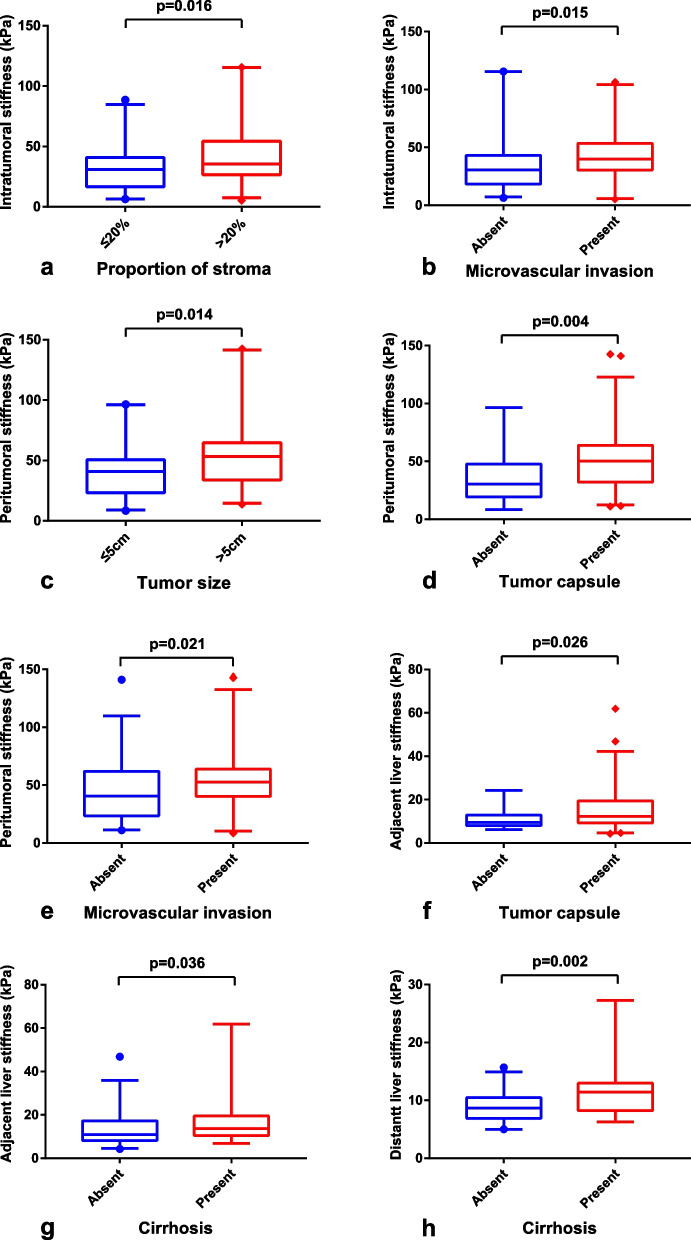
Correlations between pathological characteristics and tissue stiffness. **a**, **b** Differences in intratumoral stiffness between patients with different subgroups of stroma proportion and status of microvascular invasion. **c**–**e** Differences in peritumoral stiffness between patients with different subgroups of tumor size, tumor capsule, and status of microvascular invasion. **f**, **g** Differences in adjacent liver stiffness between patients with different subgroups of tumor capsule and cirrhosis status. **h** Differences in distant liver stiffness between patients with different cirrhosis status

Comparisons of intratumoral stiffness, peritumoral stiffness, adjacent liver stiffness, and distant liver stiffness between different pathological characteristics in 114 patients were shown in Table 2 and Fig. 3. Intratumoral stiffness was higher in tumors with the proportion of stroma of > 20% compared with tumors with the proportion of stroma of ≤ 20% (*p* = 0.016) and in tumors with MVI compared with those without MVI (*p* = 0.015). Peritumoral stiffness was higher in tumors with larger tumor size (> 5 cm, *p* = 0.014), presence of tumor capsule (*p* = 0.004), and presence of MVI (*p* = 0.021). Adjacent liver stiffness was significantly correlated with the presence of a tumor capsule (*p* = 0.026) and cirrhosis of the background liver (*p* = 0.036). While distant liver stiffness was significantly correlated with cirrhosis of background liver (*p* = 0.002).

**Table 2:** Correlations between pathological characteristics and tissue stiffness

Pathological characteristics	Intratumoral stiffness (kPa)	*p*	Peritumoral stiffness (kPa)	*p*	Adjacent liver stiffness (kPa)	*p*	Distant liver stiffness (kPa)	*p*
Tumor size		0.533		0.014		0.959		0.607
≤ 5 cm	33.9 (18.6–49.4)		40.7 (23.2–50.5)		11.3 (8.9–18.9)		8.8 (7.0–11.5)	
> 5 cm	33.6 (24.6–49.9)		53.3 (33.9–64.6)		11.8 (9.0–18.0)		9.3 (7.6–10.8)	
Proportion of stroma		0.016		0.165		0.290		0.754
≤ 20%	30.9 (16.7–40.8)		43.3 (30.2–55.2)		12.9 (9.1–20.6)		9.2 (7.5–11.7)	
> 20%	35.4 (26.5–54.2)		51.7 (28.0–66.5)		11.1 (8.7–17.4)		9.1 (7.2–10.8)	
Tumor necrosis		0.203		0.230		0.997		0.288
Absent	27.9 (17.8–50.1)		44.9 (21.7–61.2)		12.5 (8.1–19.4)		9.1 (7.9–12.8)	
Present	34.9 (25.6–49.8)		48.8 (30.2–64.7)		11.7 (9.2–18.0)		9.2 (7.2–10.6)	
Proportion of necrosis		0.158		0.205		0.454		0.828
< 20%	32.9 (20.8–44.2)		44.7 (23.9–61.9)		12.0 (9.0–19.4)		8.5 (7.3–11.4)	
≥ 20%	34.5 (26.0–55.1)		51.7 (30.1–68.2)		11.0 (8.9–17.5)		9.4 (7.0–11.0)	
Edmondson-Steiner grade		0.376		0.104		0.833		0.577
Grade I–II	30.7 (20.8–43.6)		44.3 (25.2–55.7)		12.1 (8.2–19.2)		9.1 (7.0–12.2)	
Grade III–IV	34.5 (23.1–52.1)		51.1 (30.3–67.6)		11.1 (9.2–17.7)		9.3 (7.4–10.6)	
Tumor capsule		0.602		0.004		0.026		0.497
Absent	35.3 (18.3–57.9)		30.4 (19.3–47.7)		9.5 (8.0–12.9)		8.7 (6.6–10.9)	
Present	32.9 (23.6–46.5)		50.2 (32.1–63.9)		12.3 (9.3–19.4)		9.3 (7.5–11.5)	
Peritumoral lymphocytic reaction		0.542		0.353		0.605		0.541
Low	31.3 (16.8–46.1)		43.8 (24.2–63.3)		11.9 (9.5–20.7)		8.5 (6.9–11.4)	
Moderate	32.4 (25.4–49.0)		50.6 (39.7–66.5)		11.9 (9.5–17.5)		9.3 (7.9–10.7)	
High	38.2 (22.4–54.0)		44.9 (25.3–61.0)		10.3 (8.1–18.7)		11.4 (6.6–19.4)	
Microvascular invasion		0.015		0.021		0.121		0.219
Absent	30.6 (18.3–43.2)		40.5 (23.6–61.9)		10.9 (8.6–17.5)		8.5 (7.2–10.5)	
Present	39.9 (30.4–53.4)		52.7 (40.3–63.9)		13.4 (9.3–10.9)		10.2 (7.9–11.8)	
Cirrhosis of background liver		0.777		0.050		0.036		0.002
Absent	33.7 (21.2–48.9)		43.6 (25.2–48.8)		10.9 (8.2–17.3)		8.7 (6.9–10.5)	
Present	33.4 (25.6–52.6)		54.1 (40.3–52.7)		13.7 (10.5–19.5)		11.4 (8.2–12.9)

### Quantitative 2D-SWE features and recurrence

**Fig. 4:**
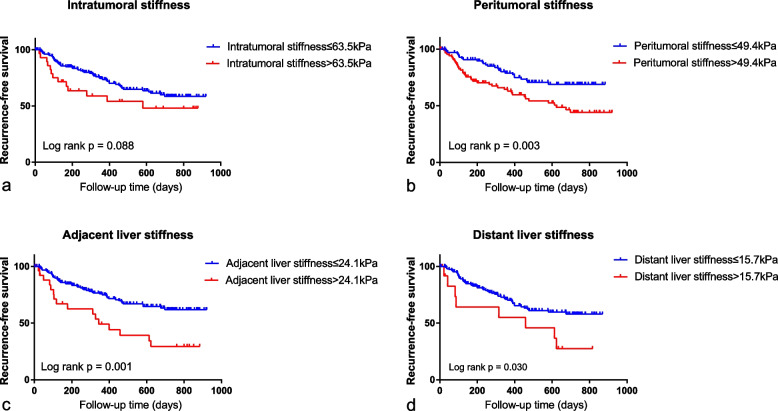
Correlations between recurrence-free survival and tissue stiffness. **a** Recurrence-free survival curves stratified by intratumoral stiffness. **b** Recurrence-free survival curves stratified by peritumoral stiffness. **c** Recurrence-free survival curves stratified by adjacent liver stiffness. **d** Recurrence-free survival curves stratified by distant liver stiffness

The optimal cut-off values of intratumoral stiffness, peritumoral stiffness, adjacent liver stiffness, and distant liver stiffness for predicting RFS were 63.5 kPa, 49.4 kPa, 24.1 kPa, and 15.7 kPa, respectively. Based on these cut-off values, patients were dichotomized into two groups and RFS was compared between the two groups. Kaplan–Meier survival analysis showed that RFS was significantly shorter in patients with higher peritumoral stiffness (*p* = 0.003), adjacent liver stiffness (*p* = 0.001), and distant liver stiffness (*p* = 0.030) compared with lower ones (Fig. 4). However, there was no significant difference in RFS between patients with high intratumoral stiffness and low intratumoral stiffness (*p* = 0.088).

### Univariable and multivariable Cox proportional hazards analyses for predictors for RFS

Univariable Cox proportional hazards analysis showed that peritumoral stiffness (HR = 2.104; 95% CI, 1.283–3.450; *p* = 0.003), adjacent liver stiffness (HR = 2.509; 95% CI, 1.428–4.408; *p* = 0.001), and distant liver stiffness (HR = 1.249; 95% CI, 1.065–4.751; *p* = 0.034) were associated with RFS (Table 3). With regard to clinical, histological and B-mode ultrasound features, tumor size (HR = 1.205; 95% CI, 1.149–1.264; *p* < 0.001), AFP level (HR = 2.078; 95% CI, 1.245–3.369; *p* = 0.005), AST level (HR = 2.119; 95% CI, 1.306–3.439; *p* = 0.002), GGT level (HR = 1.931; 95% CI, 1.164–3.206; *p* = 0.011), MVI (HR = 2.901; 95% CI, 1.768–4.762; *p* < 0.001), and Edmondson-Steiner grade (HR=1.686; 95% CI, 1.024–2.775; *p* = 0.040) were associated with RFS (Table 3).

**Table 3:** Univariate and multivariate analysis for predictors of RFS after HCC hepatectomy

Variables	Univariate analysis		Multivariate analysis	
	HR (95% CI)	*p* value	HR (95% CI)	*p* value
Sex, female vs. male	1.410 (0.693–2.955)	0.363		
Age, years	0.989 (0.968–1.010)	0.290		
Tumor size, cm	1.205 (1.149–1.264)	< 0.001	1.190 (1.125–1.258)	< 0.001
AFP, > 20 U/L vs. ≤ 20 U/L	2.078 (1.245–3.369)	0.005		
TBIL, > 17.1 µmol/L vs. ≤ 17.1 µmol/L	1.049 (0.616–1.788)	0.860		
ALB, < 35 g/L vs. ≥ 35 g/L	1.623 (0.868–3.036)	0.129		
ALT, > 40 U/L vs. ≤ 40 U/L	1.094 (0.652–1.837)	0.733		
AST, > 40 U/L vs. ≤ 40 U/L	2.119 (1.306–3.439)	0.002		
GGT, > 50 U/L vs. ≤ 50 g/L	1.931 (1.164–3.206)	0.011		
HBV-DNA, > 100 IU/mL vs. ≤ 100 IU/mL	1.454 (0.887–2.383)	0.137		
Cirrhosis, present vs. absent	0.686 (0.385–1.222)	0.201		
MVI, present vs. absent	2.901 (1.768–4.762)	< 0.001	1.764 (1.031–3.018)	0.038
Edmondson-Steiner grade, III–IV vs. I–II	1.686 (1.024–2.775)	0.040		
Shape, irregular vs. regular	1.655 (0.986–2.780)	0.057		
Boundary, unclear vs. clear	1.243 (0.665–2.325)	0.496		
Halo, present vs. absent	0.950 (0.575–1.569)	0.840		
Intratumoral stiffness, > 63.5 kPa vs. ≤ 63.5 kPa	1.685 (0.918–3.093)	0.092		
Peritumoral stiffness, > 49.4 kPa vs. ≤ 49.4 kPa	2.104 (1.283–3.450)	0.003	1.822 (1.088–3.051)	0.023
Adjacent liver stiffness, > 24.1 kPa vs. ≤ 24.1 kPa	2.509 (1.428–4.408)	0.001	1.792 (1.005–3.196)	0.048
Distant liver stiffness, > 15.7 kPa vs. ≤ 15.7 kPa	1.249 (1.065–4.751)	0.034		

*AFP*, alpha-fetoprotein; *TBIL*, total bilirubin; *ALB*, albumin; *ALT*, alanine aminotransferase; *AST*, aspartate transaminase; *GGT*, gamma-glutamyl transferase; *HBV*, hepatitis B virus; *MVI*, microvascular invasion

**Fig. 5:**
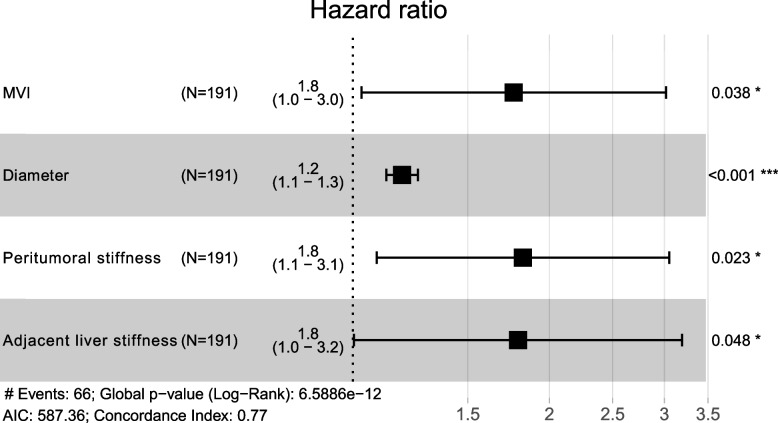
Forest plot of independent predictors of recurrence-free survival after hepatocellular carcinoma resection

Multivariable Cox proportional hazards analysis showed that larger tumor size (adjusted HR=1.190; 95% CI, 1.125–1.258; *p* < 0.001), MVI (adjusted HR = 1.764; 95% CI, 1.031–3.018; *p* = 0.038), higher peritumoral stiffness (> 49.4 kPa) (adjusted HR = 1.822; 95% CI, 1.088–3.051; *p* = 0.023), and higher adjacent liver stiffness (> 24.1 kPa) (adjusted HR = 1.792; 95% CI, 1.005–3.196; *p* = 0.048) were significant independent predictors of worse RFS (Table 3). The forest plot in Fig. 5 showed the results of multivariable Cox proportional hazards analysis.

### Development and validation of the nomogram for predicting RFS

**Fig. 6:**
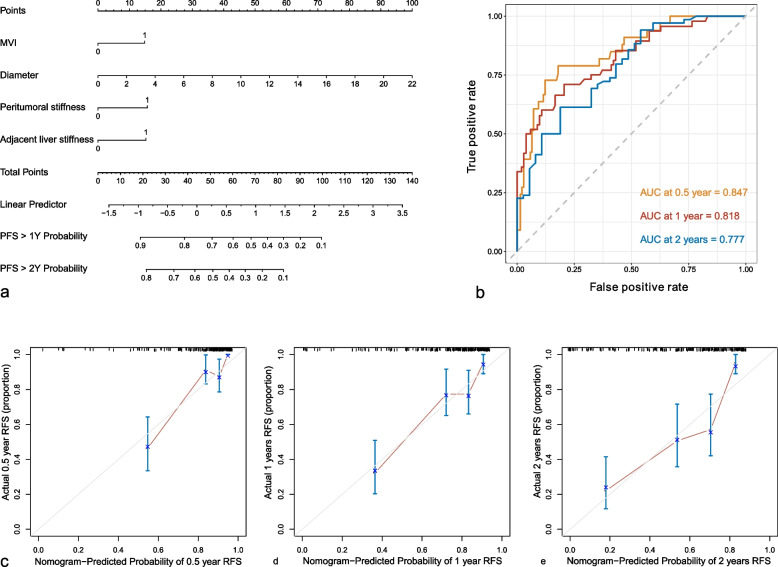
Performance of the nomogram. **a** Nomogram for predicting the recurrence-free survival in patients with hepatocellular carcinoma. **b** Receiver operating characteristic curves of the nomogram to predict 0.5-, 1-, and 2-year recurrence-free survival. **c**–**e** Calibration plots of the nomogram to predict 0.5-, 1-, and 2-year recurrence-free survival

The final nomogram integrated tumor size, MVI, peritumoral stiffness, and adjacent liver stiffness (Fig. 6a). The C-index of the nomogram was 0.77 (0.713–0.827). Receiver operating characteristic (ROC) curves to predict 0.5-, 1-, and 2-year recurrence-free RFS were shown in Fig. 6b with AUCs of 0.847, 0.818, and 0.777, respectively. The calibration curve showed good calibration for the nomogram to predict 0.5-, 1-, and 2-year recurrence-free RFS (Fig. 6c).

## Discussion

In this study, the pathological basis and prognostic implications of tumor stiffness and liver stiffness measured by 2D-SWE were explored in HBV-related HCC patients with solitary lesions and treated with surgical resection. The results indicated the important role of stiffness as an imaging biomarker of the tumor microenvironment and tumor invasiveness. Four stiffness characteristics, including intratumoral stiffness, peritumoral stiffness, adjacent liver stiffness, and distant liver stiffness, were analyzed in the current study. In HCC, higher values of the four stiffness were correlated with more aggressive metrics, such as higher proportion of stroma, presence of MVI, and larger tumor size. For the prognostic implications, peritumoral stiffness and adjacent liver stiffness were shown more valuable than intratumoral stiffness and distant liver stiffness in predicting tumor recurrence. Higher peritumoral stiffness (> 49.4 kPa) and higher adjacent liver stiffness (> 24.1 kPa) were independent risk factors for recurrence following hepatic resection in HBV-related HCC, along with traditional predictors such as larger tumor size and presence of MVI.

In this study, increased intratumor stiffness and peritumoral stiffness were shown to be associated with higher stroma proportion and the presence of tumor capsule, which were consistent with other studies showing that increased stiffness is mainly correlated with ECM remodeling and the deposition of ECM components [36, 37]. Many tumors are characterized by ECM deposition, remodeling, and cross-linking that drive fibrosis to stiffen the stroma and promote malignancy [38]. Increased stiffness can, in turn, promote numerous cellular functions that promote tumor progression and invasiveness [37]. This study also showed that intratumoral and peritumoral stiffness were correlated with the presence of MVI, a histopathologic evidence of tumor aggressiveness, which was consistent with present studies [39, 40]. These results indicated that tumor stiffness could be used as a potential biomarker to assess changes in the tumor microenvironment and tumor aggressiveness. The prognostic value of tumor stiffness was confirmed in the current study for predicting HCC recurrence after curative resection. Besides, intratumoral and peritumoral stiffness were assessed separately in this study, which was different from previous studies that integrated intratumoral and peritumoral stiffness [9–11]. Peritumoral stiffness was identified as an independent predictor of tumor recurrence. Several studies have confirmed that cancer-associated fibroblasts (CAFs), mainly located at the tumor marginal zone, enhance the production and reorganization of the ECM and lead to a mechanically stiff microenvironment [41]. Higher stiffness could promote tumor cell proliferation and invasion [5, 8]. However, our results did not show an independent prognostic value of intratumoral stiffness. This may be because that intratumoral stiffness could also be influenced by many other factors besides the stroma proportion, like the density of cancer cells and the presence of necrotic areas [36].

The microenvironment in which HCC develops also exerts a major influence on tumor development and growth [42]. This study showed that adjacent liver stiffness and distant liver stiffness were correlated with capsule status and liver fibrosis stage. Liver fibrosis is associated with an increased risk of malignancy [43]. Several studies have confirmed the role of liver stiffness in predicting HCC recurrence [13–16]. Our results showed that higher distant liver stiffness (> 15.7 kPa) was correlated with shorter RFS (*p* = 0.030), but no significant difference was found in cirrhosis presence between patients with and without recurrence. The inconsistency may be due to different cutoff values because liver stiffness ≥11 kPa is considered as cirrhosis in SuperSonic Imaging [44]. In this study, the results showed that although higher adjacent liver stiffness and higher distant liver stiffness were both related to a higher probability of recurrence, adjacent liver stiffness was shown to be an independent predictor of HCC recurrence. Periphery zones of tumor tissues are representative of tumor heterogeneity in that they are rich in highly invasive cells, which are susceptible to the formation of MVI and satellite nodules and impact postoperative recurrence [33]. Evidence also indicated that the infiltration of activated hepatic stellate cells (HSC) within adjacent liver tissues was closely associated with a poor prognosis of HCC after curative resection [45]. Activated HSCs may lead to a stiff environment and promote tumor cell dissemination [46], which may explain the prognostic value of adjacent liver tissue stiffness. The reason that distant liver stiffness did not show independent prognostic value may be that most patients were treated with antiviral therapy postoperatively, which attenuated the effect of distant liver stiffness on recurrence prediction.

A nomogram integrating tumor size, MVI, peritumoral stiffness, and adjacent liver stiffness was established with a C-index of 0.77 for RFS prediction, showing satisfactory discrimination and calibration performance. The nomogram may help stratify the risk of recurrence and improve individualized treatments and personalized surveillance strategies.

There were some limitations in this study. Firstly, there may be heterogeneity in HCC tissue while only the maximum section of the lesion was assessed. Secondly, the maximum detection depth of 2D-SWE is limited [21], but tumor elastography can still be obtained for most enrolled lesions through scanning in multiple orientations. Thirdly, quantitative elasticity measurements were acquired by a single radiologist, the generalization of the findings needs further investigation. Although the 2D-SWE approach showed high reproducibility [47], inter and intraobserver variability may affect both data acquisition and interpretation. Fourthly, only patients with hepatitis B virus-related HCC were included, so the prognostic value of the tumor stiffness and liver stiffness in other causes of underlying liver diseases and other types of liver cancer needs further study. Fifthly, this is a single-center study, and the results should be confirmed by further studies at multiple centers with larger samples.

## Conclusions

In conclusion, stiffness measured by 2D-SWE could be a useful imaging biomarker of the tumor microenvironment and tumor invasiveness in HBV-related HCC, with higher stiffness indicating more aggressive behavior. Peritumoral stiffness and adjacent liver stiffness showed important values in predicting tumor recurrence after curative resection in HBV-related HCC.

### Supplementary Information

**Additional file 1.**

## Data Availability

The datasets used and/or analyzed during the current study are available from the corresponding author on reasonable request.

## Abbreviations

2D-SWE: Two-dimensional shear wave elastography
AUC: Area under the receiver operating characteristic curve
ECM: Extracellular matrix
Emean: Mean elastic modulus
HBV: Hepatitis B virus
HCC: Hepatocellular carcinoma
MVI: Microvascular invasion
RFS: Recurrence-free survival
