# Supplementary material for: Stiffness on shear wave elastography as a potential microenvironment biomarker for predicting tumor recurrence in HBV-related hepatocellular carcinoma
Source: Insights Imaging. 2023 Sep 12;14:147. doi: 10.1186/s13244-023-01505-7 (PMC10495298; doi:10.1186/s13244-023-01505-7)
Supplement: Supplementary file 1 — Additional file 1. [file 13244_2023_1505_MOESM1_ESM.pdf]

Stiffness on Shear Wave Elastography as a Potential Microenvironment Biomarker for Predicting Tumor Recurrence in HBV-Related Hepatocellular Carcinoma

ELECTRONIC SUPPLEMENTARY MATERIAL

Definition of pathological characteristics:

Proportion of stroma: The proportion of the stroma relative to tumor cells in the overall tumor bed. All H&E slides were screened and evaluated for proportion of stroma [1]. The assessment of proportion of stroma was conducted using scoring percentages in 10% increments (10%, 20%, 30%, etc.).

The presence of tumor necrosis: Characterized by homogenous clusters of sheets of dead cells, or coalescing groups of cells forming a coagulum, containing nuclear and cytoplasmic debris on H&E slides [2].

Proportion of tumor necrosis: Proportion of necrotic tissue in the overall tumor bed. Proportion of tumor necrosis was conducted using scoring percentages in 10% increments (10%, 20%, 30%, etc.).

Tumor capsule: Fibrous capsule around the tumor [3].

Peritumoral lymphocytic reaction: Peritumoral lymphocytic reaction was defined as discrete lymphoid reactions surrounding tumor [4]. It was scored as low, moderate, or high.

Microvascular invasion (MVI): Characterized by the cancer cell nest in vessels lined

with endothelial cells [5]. Most frequently found in the small branches of the portal vein in the adjacent liver tissues.

Grade of liver fibrosis: Grade of liver fibrosis was evaluated according to Scheuer liver fibrosis staging system [6]. The fibrosis is classified into no fibrosis, mild fibrosis, moderate fibrosis, severe fibrosis, and cirrhosis.

Edmondson-Steiner grade: HCC differentiation grades were based on the Edmondson-Steiner classification [7].

References for supplementary material

1. Lv Z, Cai X, Weng X, Xiao H, Du C, Cheng J, Zhou L, Xie H, Sun K, Wu J et al: Tumor-stroma ratio is a prognostic factor for survival in hepatocellular carcinoma patients after liver resection or transplantation. Surgery 2015, 158(1):142-150.

2. Ling YH, Chen JW, Wen SH, Huang CY, Li P, Lu LH, Mei J, Li SH, Wei W, Cai MY et al: Tumor necrosis as a poor prognostic predictor on postoperative survival of patients with solitary small hepatocellular carcinoma. BMC Cancer 2020, 20(1):607.

3. Iguchi T, Aishima S, Sanefuji K, Fujita N, Sugimachi K, Gion T, Taketomi A, Shirabe K, Maehara Y, Tsuneyoshi M: Both fibrous capsule formation and extracapsular penetration are powerful predictors of poor survival in human hepatocellular carcinoma: a histological assessment of 365 patients in Japan. Ann Surg Oncol 2009, 16(9):2539-2546.

4. Ogino S, Nosho K, Irahara N, Meyerhardt JA, Baba Y, Shima K, Glickman JN, Ferrone CR, Mino-Kenudson M, Tanaka N et al: Lymphocytic reaction to colorectal

cancer is associated with longer survival, independent of lymph node count, microsatellite instability, and CpG island methylator phenotype. Clin Cancer Res 2009, 15(20):6412-6420.

5. Cong WM, Bu H, Chen J, Dong H, Zhu YY, Feng LH, Chen J: Practice guidelines for the pathological diagnosis of primary liver cancer: 2015 update. World J Gastroenterol 2016, 22(42):9279-9287.

6. Desmet VJ, Gerber M, Hoofnagle JH, Manns M, Scheuer PJ: Classification of chronic hepatitis: diagnosis, grading and staging. Hepatology 1994, 19(6):1513-1520.

7. Edmondson HA, Steiner PE: Primary carcinoma of the liver: a study of 100 cases among 48,900 necropsies. Cancer 1954, 7(3):462-503.
